# CircPRKCH modulates extracellular matrix formation and metabolism by regulating the miR-145/HGF axis in osteoarthritis

**DOI:** 10.1186/s13075-022-02893-9

**Published:** 2022-09-06

**Authors:** Wenzhong Que, Huili Liu, Qinqin Yang

**Affiliations:** 1 Department of Rheumatology, Fuzhou No. 1 Hospital Affiliated with Fujian Medical University, Taijiang District, Fuzhou, 350000 Fujian Province China; 2 Department of Medical Technology, Zhangzhou Health Vocational College, Zhangzhou, 363000 Fujian Province China; 3 College of Pharmacy, Fujian Medical University, Fuzhou, 350005 Fujian Province China

**Keywords:** CircPRKCH, Extracellular matrix, Hepatocyte growth factor, miR-145, Osteoarthritis

## Abstract

**Background:**

Osteoarthritis (OA) is a chronic degenerative joint disease. Extracellular matrix (ECM) degradation is essential for OA progression. Previous studies have shown that circular RNAs (circRNAs) are involved in the pathological process of OA. CircPRKCH has been shown to be upregulated in OA chondrocytes. The present study was aimed to explore the roles of circPRKCH in vivo and in vitro models of OA and its underlying molecular mechanisms.

**Methods:**

IL-1β-induced chondrocytes and mice injected with monosodium iodoacetate were used as OA models in vitro and in vivo, respectively. RT-qPCR was performed to measure the expression of circPRKCH, miR-145, and HGF in cartilage tissues and chondrocytes. The interaction between miR-145 and circPRKCH or HGF was verified by a dual-luciferase reporter assay. Chondrocyte apoptosis, viability, and ECM-related proteins were examined by flow cytometry, MTT assay, and Western blotting, respectively. Histopathological changes were detected by HE and Safranin O-fast green staining.

**Results:**

The expression of circPRKCH and HGF was increased in OA cartilage tissues and IL-1β-treated chondrocytes, while miR-145 expression was decreased. IL-1β induced chondrocyte apoptosis and ECM degradation in chondrocytes. Moreover, circPRKCH promoted HGF expression and activated HGF/c-MET by directly binding to miR-145. miR-145 knockdown or HGF overexpression significantly reversed circPRKCH knockdown-mediated inhibition of apoptosis and ECM degradation in IL-1β-induced chondrocytes. Besides, miR-145 overexpression alleviated IL-1β-induced chondrocyte apoptosis and ECM degradation by inhibiting HGF/c-MET. Finally, circPRKCH knockdown reduced ECM degradation by regulating the miR-145/HGF axis in an experimental OA model in mice.

**Conclusion:**

Our study demonstrated that circPRKCH promoted chondrocyte apoptosis and ECM degradation via the miR-145/HGF axis in OA, which may provide a novel target for OA treatment.

**Supplementary Information:**

The online version contains supplementary material available at 10.1186/s13075-022-02893-9.

## Background

Osteoarthritis (OA) is a prevalent degenerative joint disorder often occurring in the elderly, which is accompanied with chronic pain, swelling, and stiffness. OA is the fastest growing cause of irreversible disability worldwide, affecting ~10% of males and ~18% of females over 60 years old [1]. Progressive articular cartilage degeneration is a vital pathological factor leading to OA, due to the imbalanced anabolism and catabolism of articular cartilage [2]. During OA progression, degradation of the articular cartilage extracellular matrix (ECM) and chondrocyte apoptosis appear to increase [3]. For example, MMP-13 has been reported to be a central factor in the degradation of type II collagen during OA development [4]. In addition, cartilage destruction is also associated with various cytokines, such as IL-1β [5]. However, the underlying mechanisms involved in OA progression remain unclear [6]. Therefore, the study of key molecules in OA pathogenesis is of great significance for OA treatment.

Hepatocyte growth factor (HGF), an interstitial-derived growth factor, combines with its receptor c-MET to form a HGF/c-MET axis with a wide range of biological functions, which regulates growth and differentiation of many tissues [7]. The c-MET receptor is a transmembrane tyrosine kinase on a variety of cell types such as chondrocytes [8]. A previous report demonstrated that HGF played a vital role in OA cartilage [9]. HGF has been confirmed to modulate Leydig cell ECM components [10]. Therefore, we hypothesized a role for the HGF/c-MET pathway during OA progression.

MicroRNAs (miRNAs) are a class of small non-coding RNAs consisting of 18–24 nucleotides and act as gene regulators in many diseases [11]. MiRNAs have been found to play critical roles in OA-related pathology [12]. For instance, miR-127-5p regulated MMP-13 expression in human chondrocytes [13]. Wu et al. showed that miR-449a overexpression could promote chondrocyte ECM degradation in OA [14]. It has been illuminated that miR-145 reduced cartilage matrix degradation in OA by directly inhibiting MKK4 signaling [15]. Moreover, miR-145 has been shown to regulate IL-1β-induced cartilage degradation in human chondrocytes [16]. In addition, a previous in vivo experiment indicated that miR-20b suppressed OA progression through regulating PTEN in an OA mouse model [17]. However, the underlying mechanism of miR-145 in the pathological process of OA requires further investigation.

Circular RNAs (circRNAs), a type of non-coding RNAs, form a stable ring structure with covalent bonds and regulate gene expression [18]. Researchers consider circRNAs key regulators of the inflammatory response induced by OA [19]. Interaction between circRNA and miRNA is considered as an important factor in OA. For instance, Wu et al. reported that circRNA hsa_circ_0005105 served as a miR-26a sponge and regulated the expression of NAMPT during ECM degradation in OA [20]. Zhou et al. showed that circRNA Atp9b induced OA progression via targeting miR-138-5p [21]. Therefore, a deeper understanding of the mechanisms of circRNAs in OA will facilitate the development of novel therapies. According to some reports, circPRKCH (ID: hsa_circ_0032131), which is derived from exons 11–12 of the PRKCH gene on chromosome chr14 with a length of 328 bp, was significantly increased in cartilage tissue of OA [22]. Moreover, high expression of circPRKCH in peripheral blood could be used as a potential diagnostic biomarker for OA [23]. Nevertheless, the role of circPRKCH in OA progression is still uncovered. CircPRKCH and HGF were predicted to be targets of miR-145 based on bioinformatic analysis. Thus, we proposed that circPRKCH might promote ECM degradation and lead to OA occurrence through targeting miR-145 and activating the HGF/c-MET signaling pathway.

The present study focused on the promotion of circPRKCH in OA development and found that circPRKCH was highly expressed in OA tissue specimens and IL-1β-treated chondrocytes. In addition, circPRKCH silencing was revealed to inhibit chondrocyte apoptosis and ECM degradation via miR-145, thus suppressing the HGF/c-MET signaling pathway. These findings demonstrated a potential mechanism of circPRKCH in OA development, and circPRKCH may be a key regulator and therapeutic target for OA.

## Methods

### Patients and tissue samples

Cartilage samples were obtained from 16 OA patients (7 men and 9 women, age range 54-72 years) who underwent total knee replacement at the Affiliated Nanping First Hospital of Fujian Medical University. Normal articular cartilage samples were isolated from 10 donors (5 men and 5 women, age range 45–67 years), who underwent trauma without a history of OA or rheumatoid arthritis. All cartilage samples were immediately frozen and stored in liquid nitrogen. The diagnosis of OA patients was made according to the criteria of the American College of Rheumatology. OA severity was graded using weight-bearing anteroposterior radiographs of the affected knee.

### Cell culture and treatment

Human primary chondrocytes were isolated from cartilage tissues as previously described [24]. In brief, cartilage tissues were firstly digested with 0.25% trypsin (Invitrogen, Carlsbad, CA, USA) for 30 min, followed by 0.2% type II collagenase (Invitrogen) for 4 h at 37°C. The 293T cells were purchased from the American Type Culture Collection (ATCC, Manassas, VA). The obtained chondrocytes and 293T cells were cultured in Dulbecco’s modified Eagle medium (DMEM) (Thermo Fisher Scientific, Waltham, MA, USA), with 10% fetal bovine serum (Invitrogen), 1% penicillin-streptomycin (Sigma, St. Louis, MO, USA), and 1% l-glutamine (Invitrogen) at 37°C and 5% CO_2_ in a humidified atmosphere. The chondrocytes were stimulated with 10 ng/ml of IL-1β (Sigma-Aldrich, St. Louis, MO, USA) for 12 h.

### Cell transfection

Lentiviral particles expressing sh-circPRKCH or HGF were designed and synthesized by GenePharma (Shanghai, China). The miR-145 mimics, miR-145 inhibitor, and negative controls were purchased from RiboBio (Guangzhou, China). When cells grew to 60% confluence, the transfection was performed using Lipofectamine 2000 reagent (Invitrogen). Cells were harvested after 48 h. After at least 1 week of viral infection, stable cell clones could be selected with antibiotics (puromycin, 2–5μg/ml, Sigma).

### MTT assay

Cell viability was assayed by MTT assay. The cultured chondrocytes were inoculated in 96-well plates (1×10^5^ cells/well). Then, 20 μl (500 μg/ml) MTT solution (Sigma) was added to each well at 37°C for 4 h. After MTT removing, cells were mixed with 150 μl of dimethyl sulfoxide (DMSO, Sigma) for 10 min. A microplate reader (Bio-Rad Laboratories, Inc., Hercules, CA, USA) was used to detect the optical density at a wavelength of 490 nm.

### Flow cytometry assay

Cell apoptosis was detected using the Annexin V-FITC/propidium iodide (PI) apoptosis kit (Invitrogen). Briefly, chondrocytes were cultured for 48 h, digested using trypsin without EDTA (Thermo Fisher Scientific), and washed with PBS. The cells were collected and adjusted to a cell density of 1 × 10^6^ cells/ml. Then, cells were stained with Annexin V-FITC and PI for 15 min. At last, a BD FACS flow cytometer (BD Biosciences, Franklin Lakes, NJ, USA) was used to detect the fluorescence intensity.

### Dual-luciferase reporter assay

Artificially synthesized circPRKCH wild-type and mutant-type (namely circPRKCH-WT/MUT) and HGF 3′-UTR wild-type and mutant-type (HGF 3′-UTR-WT/MUT) constructs containing the putative binding sites of miR-145 (GenePharma, Shanghai, China) were inserted into psiCHECK2 vectors (Promega, Madison, WI, USA). Lipofectamine 2000 (Invitrogen) was then used to co-transfect miR-145 mimics or NC mimics with wild-type or mutant constructs into 293T cells. Forty-eight hours after transfection, luciferase activity was detected using the luciferase reporter gene analysis system (Promega).

### Animal experiments

Specific-pathogen-free (SPF) grade male C57BL/6 mice (8 weeks old; 18–22 g) were purchased from SJA Laboratory Animal Co., Ltd (Hunan, China, *n*=20), which were housed in ventilated racks at 21–22 °C with a 12-h light/12h dark cycle. Sterile food and water were provided. OA was induced by injecting monosodium iodoacetate into the right knee joint of mice. Briefly, after deep anesthetization with isoflurane (2–4 %), mice were injected with monosodium iodoacetate (Sigma-Aldrich) (3 mg in saline). Control mice were injected with an equivalent volume of PBS. As described previously [17], the mice were randomly divided into four groups: control, OA, OA+sh-NC, and OA+sh-circPRKCH (*n*=5 each group). Lentiviral particles expressing shRNA targeting circPRKCH were injected into the mouse tail vein. The OA mice in the sh-NC group were injected with empty lentiviral particles in the same way. After 8 weeks of operation, all mice were sacrificed by CO_2_ inhalation. Left knee joint tissues were separated and processed for further experiments.

### Histological examination

The cartilage tissue specimens were fixed in 4% paraformaldehyde for 24 h. The specimens were dehydrated and embedded in paraffin. Tissue blocks were then sliced into sections (5 μm thick). After that, the sections were deparaffinized in xylene and rehydrated with graded ethanol. Then, the sections were stained with hematoxylin for 5 min, followed by immersing in 1% acidic ethanol (1% HCl in 70% ethanol) for 20 s and rinsing with distilled water for 2 min. Subsequently, the sections were stained with eosin solution for 3 min, dehydrated, and mounted. The slices were photographed with an Olympus light microscope (Olympus, Tokyo, Japan).

To assess cartilage destruction, each section was stained with Safranin O-Fast Green solution (Sigma) for 4 min. After being washed in distilled water, sections were dehydrated in graded ethanol and mounted from xylene. Pathological changes were observed under a microscope (Olympus).

### RNA extraction and real-time quantitative PCR

Trizol reagent (Invitrogen) was used to extract total RNA. PrimeScript™ RT Kit (Takara, Kyoto, Japan) was used for reverse transcription of circPRKCH and HGF. Reverse transcription of miR-145 was conducted using a Mir-X^TM^ miRNA First Strand Synthesis Kit (Takara). Glyceraldehyde-3-phosphate dehydrogenase (GAPDH, for circPRKCH and HGF) and U6 small nuclear RNA (U6, for miR-145) were used as internal controls. The qPCR was conducted using the SYBR Green PCR Kit (Takara). Relative quantitative analysis of gene expression was calculated by the standard 2^-∆∆Ct^ method. The following primers: circPRKCH F: 5′-GTCTACCCTACCTGGCTCCAT-3′, R: 5′-CATTGCAAATCCCCTCCTTGC-3′; miR-145 F: 5′-TCCAGTTTTCCCAGGAATCCCT-3′, R: 5′-CGCTTCACGAATTTGCGTGTCAT-3′; HGF F: 5′-AAATCCTCGAGGGGAAGAAG-3′, R: 5′-AGCCCTTGTCGGGATATCTT-3′; GAPDH F: 5′-CATCATCCCTGCCTCTACTGG-3′, R: 5′-GTGGGTGTCGCTGTTGAAGTC-3′; U6 F: 5′-CTCGCTTCGGCAGCACA-3′, R: 5′-AACGCTTCACGAATTTGCGT-3′. The data were analyzed using the standard 2^-∆∆Ct^ method. The experiment was performed in triplicate.

### Western blot assay

Cells were lysed and protein was extracted using RIPA buffer (Invitrogen). In addition, the BCA protein assay kit (Beyotime Bio, Shanghai, China) was used to detect the concentration of protein. Equal amounts of protein (30 μg) samples were separated using 10% SDS-PAGE gels and then transferred to PVDF membranes (Millipore, Billerica, MA, USA). After blocking with 5% skim milk, the membranes and the following primary antibodies were incubated overnight at 4°C: anti-HGF (EPR12230, 1:1000, Abcam, Cambridge, MA, USA), anti-MMP-3 (EP1186Y, 1:1000, Abcam), anti-MMP-13 (1: 2000; Abcam), anti-Aggrecan (6-B-4, 1:1000, Abcam), anti-Collagen II (EPR12268, 1:1000, Abcam), anti-c-MET (EPR19067, 1:1000, Abcam), anti-p-c-MET (EP2367Y, 1:1000, Abcam), and GAPDH (6C5, 1:2000, Abcam). GAPDH protein was used as the inner control. After washing, the membranes were incubated with the HRP-conjugated secondary antibody at 1:2000 dilution for 1 h at room temperature. The resulting bands were detected with an ECL detection system (Millipore).

### Statistical analysis

Graphpad Prism 7.0 software (Graphpad, La Jolla, CA, USA) was executed to analyze all data. In this study, each experiment was repeated independently at least three times. Data were presented as mean ± standard deviation (SD). Statistical differences between the two groups were assessed by Student’s *t* test. In addition, a one-way analysis of variance (ANOVA) followed by Tukey post hoc was carried out for multiple group comparisons. When *P* < 0.05, it was considered to be statistically significant.

## Results

### The expression of circPRKCH and HGF was increased, while miR-145 was decreased in OA cartilage tissues

**Fig. 1:**
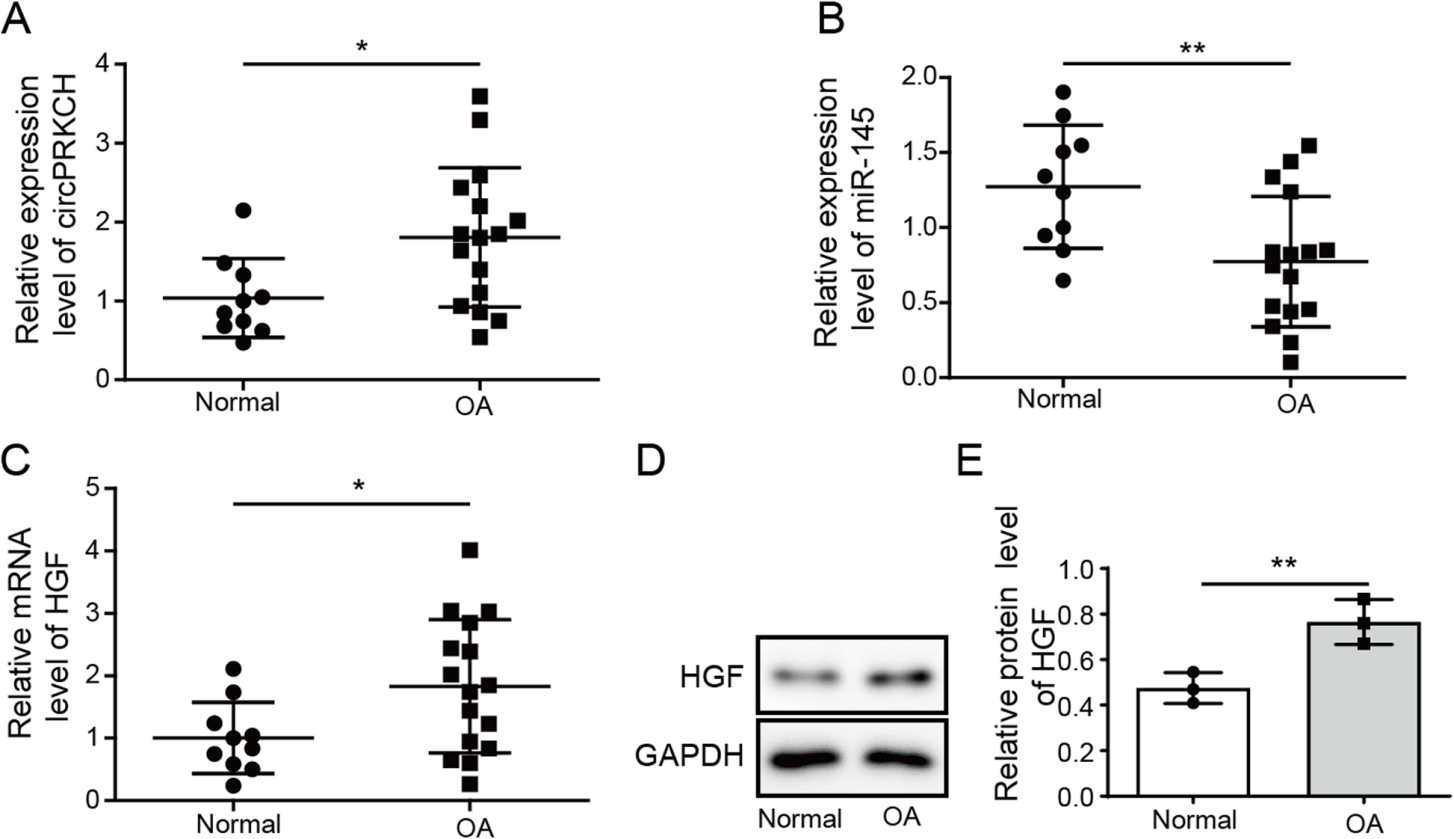
CircPRKCH and HGF were increased, but miR-145 was decreased in OA cartilage tissues. **A**–**C** RT-qPCR was used to detect circPRKCH, miR-145, and HGF expression in cartilage tissues of OA patients (*n*=16) and normal patients (*n*=10). **D**, **E** Western blotting was used to test HGF levels in OA patients and normal patients. Data are from three independent experiments. * *p*< 0.05 and ** *p*< 0.01

We first detected the expression of circPRKCH, miR-145, and HGF in OA patients (*n*=16) and normal patients (*n*=10) by RT-qPCR. The results revealed that circPRKCH and HGF were highly expressed in cartilage tissues of OA patients compared to control patients, while miR-145 expression was decreased (Fig. 1A–C). Additionally, Western blotting determined the protein level of HGF in OA patients and normal controls. Similarly, the protein level of HGF in cartilage tissues of OA patients was increased compared with normal patients (Fig. 1D, E). The above results implied that the aberrant expression of circPRKCH, miR-145, and HGF might be associated with OA.

### CircPRKCH and HGF were increased, but miR-145 was decreased in IL-1β-treated chondrocytes

**Fig. 2:**
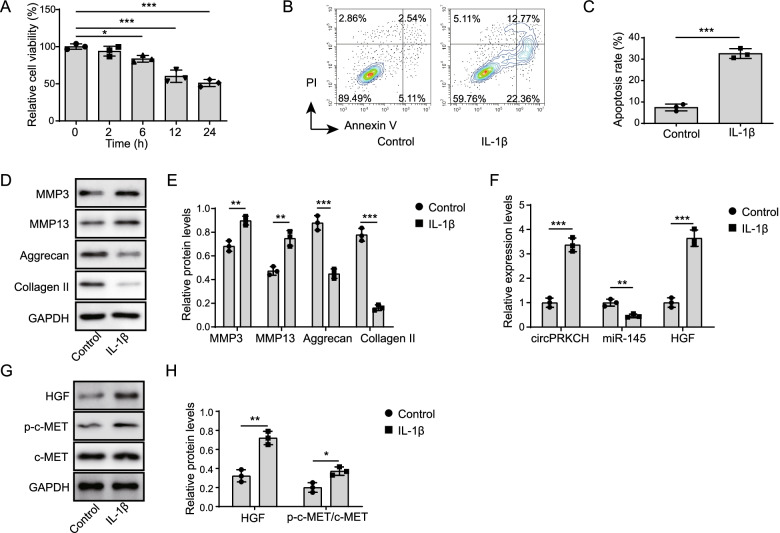
CircPRKCH and HGF were increased, but miR-145 was decreased in IL-1β-treated chondrocytes. Primary human chondrocytes were isolated and treated with 10 ng/ml IL-1β to induce an OA cell model. **A** MTT assay was used to examine chondrocyte viability in different time points. **B**, **C** Flow cytometry was used to analyze chondrocytes apoptosis. **D**, **E** Western blotting was used to detect MMP-3, MMP-13, Aggrecan, and Collagen II expression. **F** RT-qPCR was used to detect the expression levels of circPRKCH, miR-145, and HGF levels. **G**, **H** Western blotting was used to test the protein levels of HGF, p-c-MET, and c-MET. Data are the means ± SD for three independent experiments. *p< 0.05, **p< 0.01, and ***p< 0.001

To study the effect of circPRKCH in vitro, the primary human chondrocytes were further isolated and treated with 10 ng/ml IL-1β to construct an OA cell model. MTT assay demonstrated that IL-1β could inhibit chondrocyte viability in a time-dependent manner (Fig. 2A). After 12 h of treatment, the viability of chondrocytes decreased by approximately 50%. Therefore, this time point was chosen for subsequent experiments. Meanwhile, a flow cytometric assay revealed that IL-1β facilitated the apoptosis of chondrocytes compared with the control group (Fig. 2B, C). To explore the role of IL-1β in ECM degradation, Western blotting detected the levels of matrix metalloproteinase 3 (MMP-3), matrix metalloproteinase 13 (MMP-13), Aggrecan and Collagen II. The expression of Aggrecan and Collagen II in IL-1β-treated chondrocytes was obviously decreased (Fig. 2D, E). In contrast, MMP-3 and MMP-13 expression was significantly increased with IL-1β treatment (Fig. 2D, E). We further analyzed circPRKCH, miR-145, and HGF levels in chondrocytes treated by IL-1β. Consistent with data from patient cartilage tissues, IL-1β promoted circPRKCH and HGF expression in chondrocytes, but inhibited miR-145 expression (Fig. 2F–H). Moreover, the protein level of phosphorylated c-MET in IL-1β-induced chondrocytes was increased compared with the control group (Fig. 2G, H), suggesting that IL-1β activated the HGF/c-MET signaling pathway in chondrocytes. Taken together, these results implied that abnormal expression of circPRKCH and miR-145 was related to chondrocyte apoptosis and ECM degradation.

### CircPRKCH promoted HGF expression and activated the HGF/c-MET signaling pathway by directly binding to miR-145

**Fig. 3:**
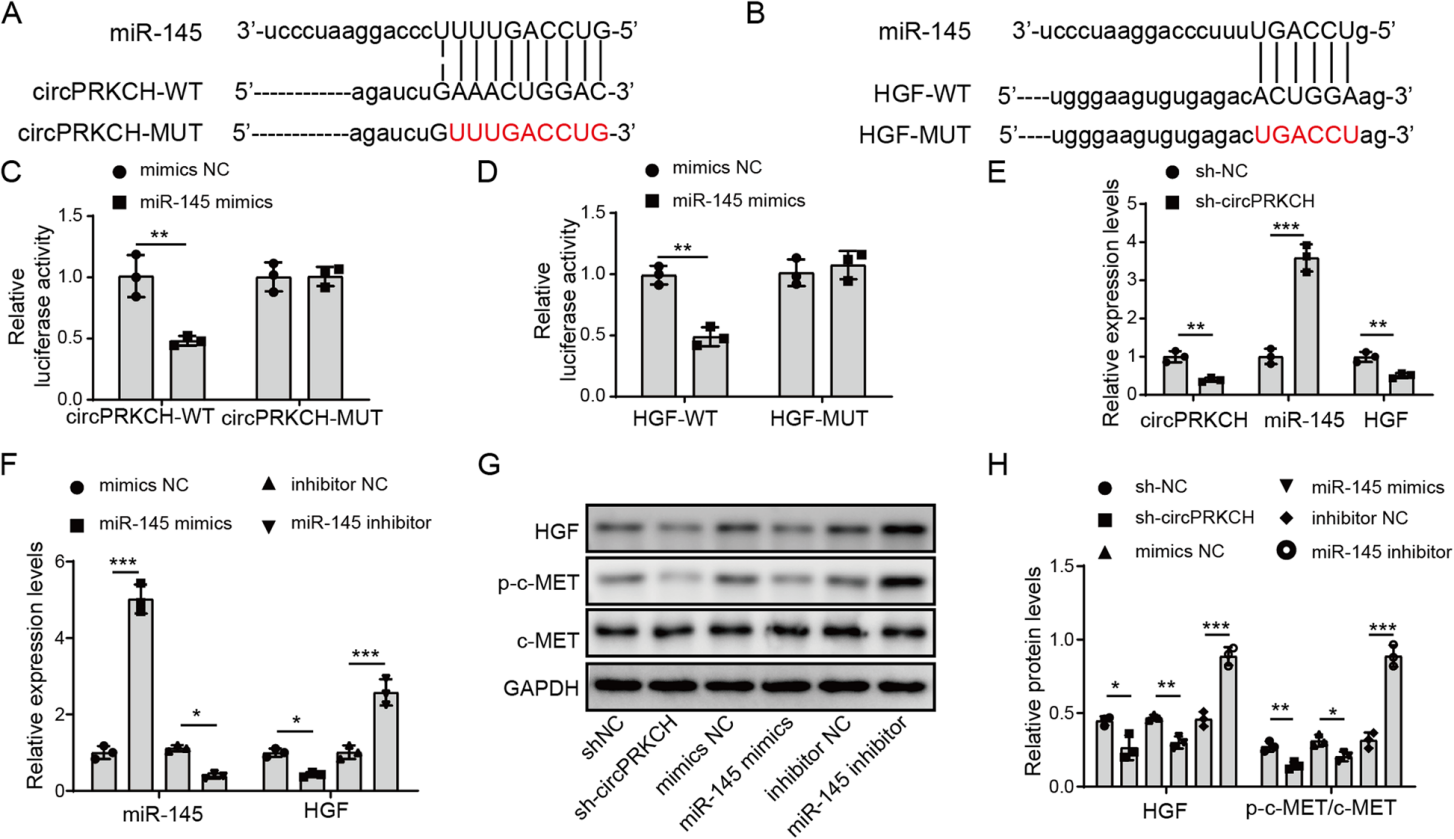
CircPRKCH activated the HGF/c-MET signaling pathway by targeting miR-145 in chondrocytes. **A**, **B** The binding sites between circPRKCH and miR-145, and miR-145 and HGF predicted by bioinformatics tools StarBase and Targetscan, respectively. **C**, **D** The luciferase activity of wild-type (WT-circPRKCH, WT-HGF) and mutant (MUT-circPRKCH, MUT-HGF) co-transfected with miR-145 mimics or mimics NC in chondrocytes. **E** Chondrocytes were transfected with sh-circPRKCH or sh-NC. RT-qPCR measured circPRKCH, miR-145, and HGF levels. **F** Chondrocytes were transfected with miR-145 mimics, miR-145 inhibitor, or negative controls. MiR-145 and HGF expression was detected by RT-qPCR. **G**, **H** Chondrocytes were treated with sh-circPRKCH, miR-145 mimics, or miR-145 inhibitor. Western blotting was used to test HGF, p-c-MET, and c-MET expression. Data are the means ± SD for three independent experiments. *p< 0.05, **p< 0.01, and ***p< 0.001

To further investigate whether or not circPRKCH acts as a ceRNA in OA, we used bioinformatics tools (StarBase and Targetscan) to predict potential interactions. We discovered one conserved binding site for miR-145 in circPRKCH (Fig. 3A). There was also one potential binding site in HGF that could interact with miR-145 (Fig. 3B). To confirm this hypothesis, luciferase reporter plasmids were constructed with wild-type binding site or mutant site in circPRKCH (Fig. 3A). Besides, the 3′-UTR of HGF containing miR-145 binding site (WT-HGF) or mutant site (MUT-HGF) was inserted into plasmids (Fig. 3B). The plasmids were then co-transfected with miR-145 mimics or mimics NC into 293T cells. Co-transfection of miR-145 mimics with WT-circPRKCH or WT-HGF significantly reduced luciferase activity. However, no obvious changes were observed for circPRKCH-MUT or HGF-MUT (Fig. 3C, D). Next, sh-circPRKCH or negative control was transfected into chondrocytes. RT-qPCR result showed that sh-circPRKCH transfection led to a decrease of circPRKCH expression (Fig. 3E). Furthermore, increased miR-145 level was observed after treatment with sh-circPRKCH or miR-145 mimics, while its expression was decreased after treatment with miR-145 inhibitor (Fig. 3E, F). Knockdown of circPRKCH or overexpression of miR-145 inhibited HGF expression, while miR-145 inhibitor promoted HGF expression (Fig. 3E, F). Simultaneously, sh-circPRKCH or miR-145 mimics acted as inhibitors of the HGF/c-MET axis to downregulate the protein expression of HGF and phosphorylated c-MET, but the miR-145 inhibitor had an opposite effect (Fig. 3G, H). Therefore, these results demonstrated that circPRKCH activated the HGF/c-MET signaling pathway via targeting miR-145 in chondrocytes.

### Knockdown of circPRKCH inhibited IL-1β-induced chondrocyte apoptosis and ECM degradation by targeting miR-145

**Fig. 4:**
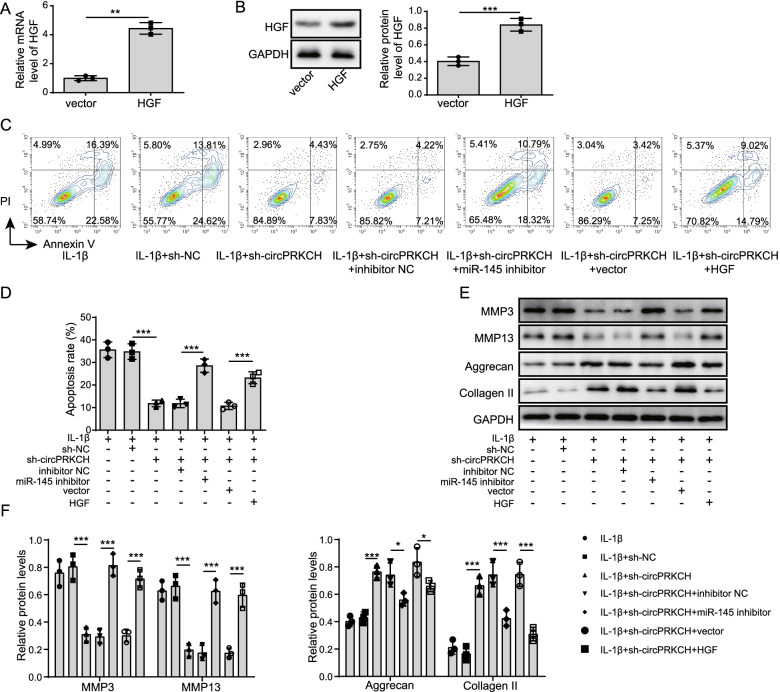
MiR-145 inhibitor or HGF overexpression reversed the effect of circPRKCH knockdown on cell apoptosis and ECM degradation. Chondrocytes were transfected with sh-circPRKCH, miR-145 inhibitor, HGF overexpressing vector, and negative controls and treated with IL-1β. **A**, **B** RT-qPCR and Western blotting were used to detect HGF level in chondrocytes. **C**, **D** Flow cytometry was used to analyze chondrocyte apoptosis. **E**, **F** Western blotting was used to determine MMP-3, MMP-13, Aggrecan, and Collagen II levels. Data are the means ± SD for three independent experiments. *p < 0.05, **p < 0.01, and ***p < 0.001

To explore the contribution of circPRKCH to OA development, IL-1β-treated chondrocytes were transfected with sh-circPRKCH, miR-145 inhibitor, or HGF overexpressing vector and the negative controls. Increased expression of HGF was observed in HGF overexpressing cells (Fig. 4A, B). Additionally, flow cytometry demonstrated that sh-circPRKCH suppressed IL-1β-induced chondrocyte apoptosis, while co-transfection of miR-145 inhibitor or HGF overexpression reversed the effect of circPRKCH knockdown on chondrocyte apoptosis (Fig. 4C, D). Simultaneously, a Western blot assay showed that knockdown of circPRKCH inhibited IL-1β-induced ECM degradation, but the inhibitory effect was overturned by co-transfection of miR-145 inhibitor or HGF overexpression (Fig. 4E, F). The above results indicated that miR-145 inhibitor or HGF overexpression reversed the effect of circPRKCH silencing on cell apoptosis and ECM degradation in IL-1β-induced chondrocytes.

### Overexpression of miR-145 inhibited IL-1β-induced degradation of ECM through inhibiting the HGF/c-MET signaling pathway

**Fig. 5:**
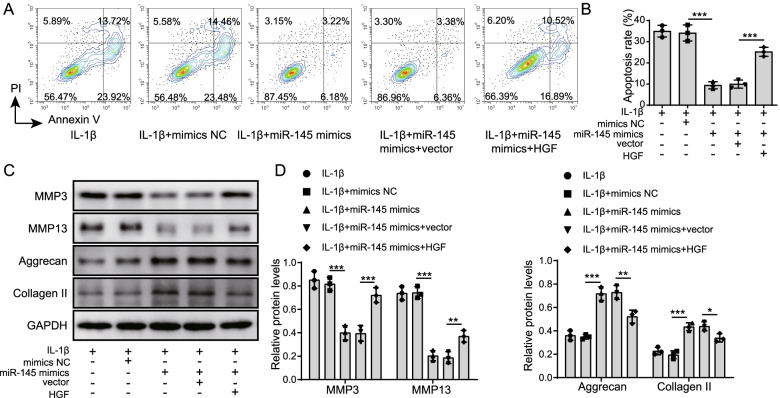
MiR-145 overexpression inhibited ECM degradation by inhibiting the HGF/c-MET signaling pathway. Chondrocytes were transfected with miR-145 mimics or HGF overexpressing vector and treated with IL-1β. **A**, **B** Flow cytometry was used to determine chondrocyte apoptosis. **C**, **D** Western blot assay was used to measure MMP-3, MMP-13, Aggrecan, and Collagen II levels. Data are the means ± SD for three independent experiments. **p< 0.01 and ***p< 0.001

To further evaluate the effect of miR-145 on OA development, IL-1β-induced chondrocytes were transfected with miR-145 mimics or HGF overexpression. Flow cytometry results showed that overexpression of miR-145 inhibited IL-1β-induced chondrocyte apoptosis, whereas co-transfection of HGF overexpression reversed the above changes (Fig. 5A, B). Consistently, the MMP-3 and MMP-13 levels in IL-1β-induced chondrocytes with miR-145 overexpression were significantly reduced, but the expressions of Aggrecan and Collagen II were greatly elevated. Following HGF overexpression, opposite results were observed (Fig. 5C, D). Our data suggested that miR-145 overexpression inhibited IL-1β-induced ECM degradation through inhibiting the HGF/c-MET signaling pathway.

### Knockdown of circPRKCH alleviated ECM degradation in the OA mouse model by regulating the miR-145/HGF axis

**Fig. 6:**
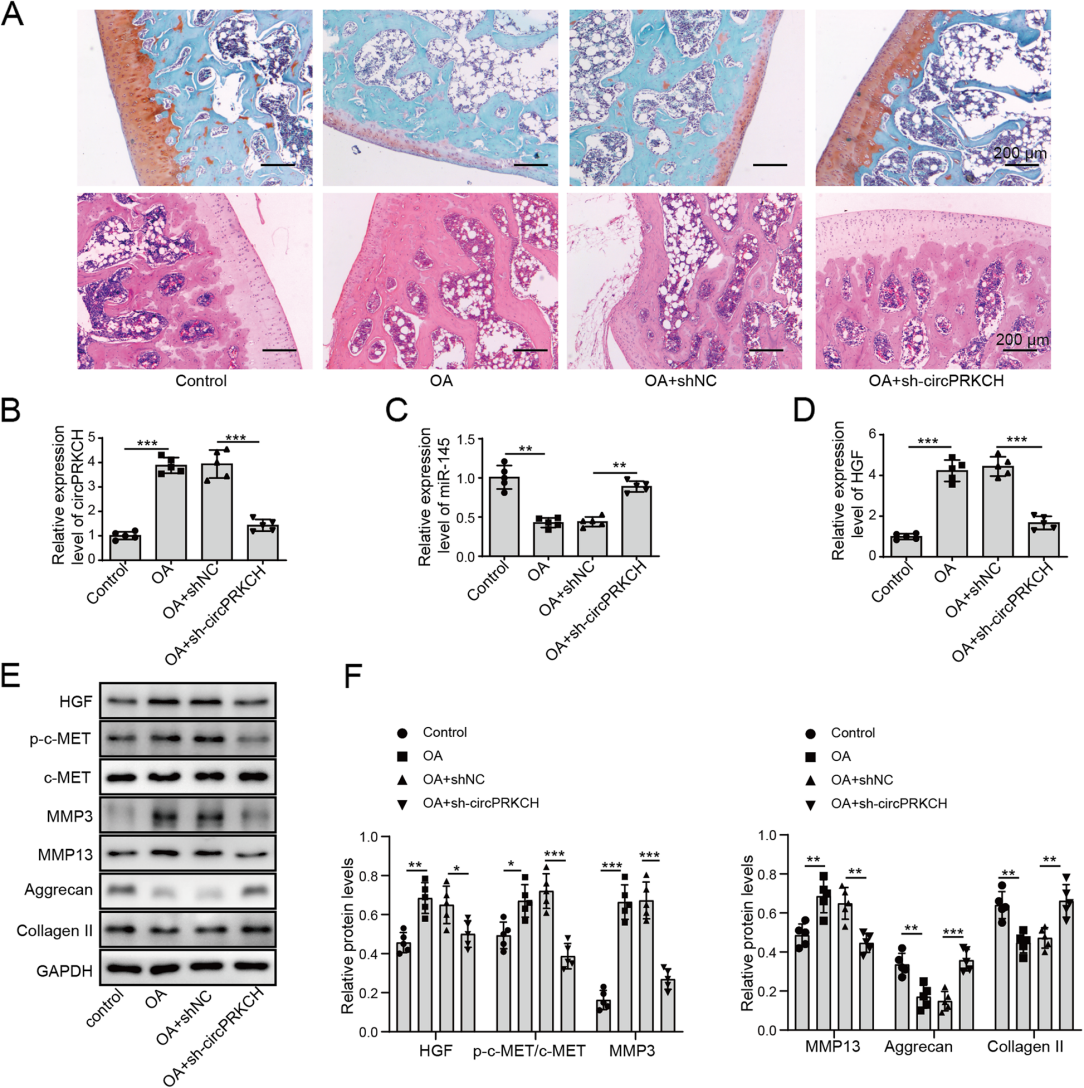
Knockdown of circPRKCH alleviated ECM degradation in an OA mouse model by regulating the miR-145/HGF axis. An OA mouse model was established by injecting sodium iodoacetate into the joint cavity of mice. Four groups were divided: Control, OA, OA+sh-NC, and OA+sh-circPRKCH. **A** HE and Safranin O-Fast Green staining were used to evaluate the osteoarthritis pathological changes and cartilage destruction. **B**–**D** RT-qPCR was used to detect circPRKCH, miR-145, and HGF levels. **E**, **F** Western blotting was used to measure HGF, p-c-MET, c-MET, MMP-3, MMP-13, Aggrecan, and Collagen II levels in mouse cartilage tissues. Data are the means ± SD for three independent experiments. *p < 0.05, **p < 0.01, and ***p < 0.001

We next explored whether circPRKCH silencing had a protective function in OA in vivo. An OA mouse model was constructed by injecting sodium iodoacetate into the joint cavity of mice. ShRNA lentiviral particles targeting circPRKCH were injected into OA mice to inhibit circPRKCH expression. HE and Safranin O-Fast Green staining were performed to assess the pathological changes. Compared with the sham group, the boundary between the cartilage and the subchondral bone (adhesive line) and the tidal line were not clearly displayed in OA mice. The chondrocytes were strikingly diminished and formed a disordered layer. In the meantime, reduced glycosaminoglycan content in the cartilage matrix and increased amount of fiber were observed in OA mice (Fig. 6A). However, knockdown of circPRKCH ameliorated these pathological changes and cartilage destruction in OA mice obviously (Fig. 6A). As expected, circPRKCH and HGF expression was increased remarkably in OA cartilage, while miR-145 was decreased. Most importantly, the knockdown of circPRKCH reversed these effects (Fig. 6B–D). In addition, in OA mice, the protein levels of HGF, phosphorylated c-MET, MMP-3, and MMP-13 were apparently increased, and the expression of Aggrecan and Collagen II were dramatically decreased, whereas these effects were abolished by circPRKCH knockdown (Fig. 6E, F). The above results indicated that circPRKCH silencing alleviated ECM degradation by regulating the miR-145/HGF axis in vivo.

## Discussion

**Fig. 7:**
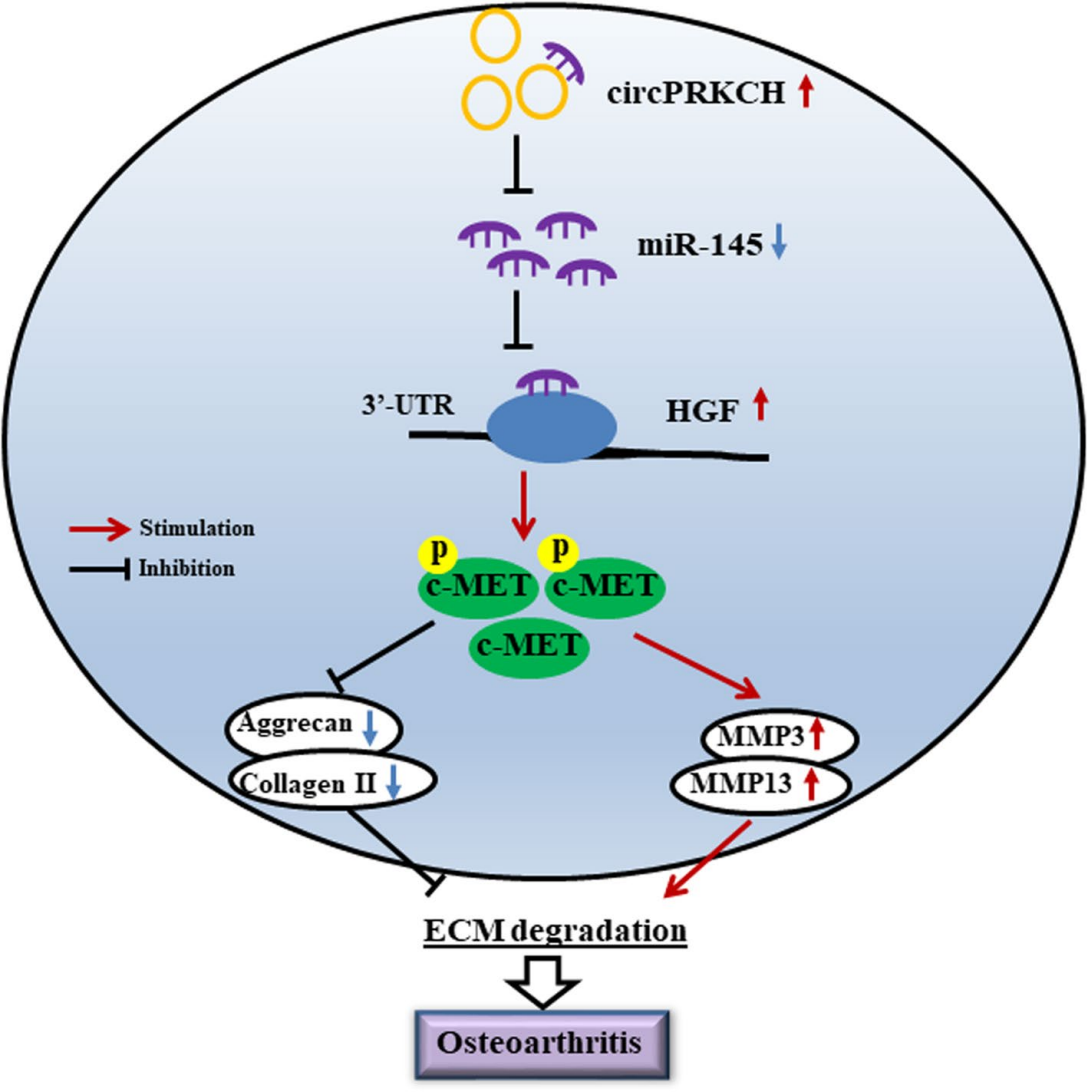
The schematic diagram of circPRKCH regulation mechanism in OA. CircPRKCH promoted c-MET phosphorylation and activated the HGF/c-MET signaling pathway by directly binding miR-145 to disinhibit HGF, leading to ECM degradation and OA occurrence

OA is a common degenerative disease among senior people and often leads to joint dysfunction [25]. The main pathological feature is articular cartilage degeneration caused by ECM degradation and chondrocyte reduction [26]. Matrix metalloproteinases such as MMP-3 and MMP-13 play key roles in decreasing Aggrecan and Collagen II, contributing to the articular cartilage degeneration during the development and progression of OA [27, 28]. Recently, metabolic activation of chondrocytes appears to be a potentiality treatment for OA [29]. Here, we demonstrated that circPRKCH silencing significantly inhibited ECM degradation and chondrocytes apoptosis in vitro and in vivo by suppressing the HGF/c-MET signaling pathway via targeting miR-145 (Fig. 7). Our data suggested that circPRKCH might be used as a potential biomarker or therapeutic target for the treatment of OA.

CircRNAs are a newly discovered class of non-coding RNA and function as a miRNA sponge that interacted with RNA or protein to regulate gene transcription [18]. Accumulating evidence suggested that multiple circRNAs were abnormally expressed in the pathological process of OA. For example, Zhu et al. found that circGCN1L1 triggered chondrocyte apoptosis via miR-330-3p and TNF-α in TMJ osteoarthritis [30]. Liu et al. revealed that circRNA regulated MMP-13 expression via targeting miR-136 during cartilage degradation [31]. CircPRKCH was found to be highly expressed in cartilage tissue and peripheral blood of OA patients [22, 23]. In accordance with these studies, we found that circPRKCH was obviously increased in OA cartilage tissues and IL-1β-treated chondrocytes. Moreover, circPRKCH silencing impaired cell apoptosis and ECM degradation. In addition, our data demonstrated that circPRKCH silencing alleviated the pathological damage in the OA mouse model, resulting in an obvious loss of MMP-3 and MMP-13 expression.

Recent studies illuminated that miRNAs played a crucial role in OA development. Chen et al. showed that miR-15a-5p regulated cell viability and matrix degradation in chondrocytes through targeting VEGFA [32]. CircRNA is known to act as a miRNA sponge to modulate gene transcription [33]. In this study, we further expanded the target miRNAs of circPRKCH. We utilized bioinformatic analysis to predict that circPRKCH possessed binding sites for miR-145, a miRNA that is located at chromosome 5q32 and interacts with multiple circRNAs. For instance, Zong et al. showed that circWHSC1 induced ovarian cancer progression via sponging miR-145 [34]. Moreover, circPVT1 promoted metastasis through miR-145 in colorectal cancer [35]. MiR-145 was also found to suppress ECM degradation in OA through suppressing MKK4 [15]. In the present study, miR-145 was down-regulated in OA cartilage tissues and IL-1β-treated chondrocytes. CircPRKCH silencing alleviated IL-1β-induced chondrocyte apoptosis and ECM degradation through miR-145. Also, we presented that overexpression of miR-145 suppressed IL-1β-induced chondrocyte apoptosis and ECM degradation. Similarly to our results, a recent study demonstrated that miR-145 modulated chondrocyte viability and cartilage matrix degradation through ADAMTS5 in OA [36]. Our data suggested that miR-145 was a key downstream factor of circPRKCH-mediated OA progression.

HGF has been reported to be involved in chondrocyte proliferation and proteoglycan synthesis [37]. Kwiecinski et al. demonstrated that HGF suppressed collagen I and IV synthesis in hepatic stellate cells [38]. HGF was shown to promote both type II collagen synthesis and alkaline phosphatase activity in chondrocytes [39]. A previous study has suggested that HGF may contribute to altered metabolism in OA cartilage [9]. However, the underlying mechanism is unclear. Indeed, we identified that HGF acted as a sponge of miR-145. More convincingly, circPRKCH knockdown or miR-145 overexpression inhibited ECM degradation in chondrocytes by targeting HGF to inhibit the c-MET signaling pathway, and these findings strongly indicated that the circPRKCH/miR-145/HGF axis modulated the extracellular matrix metabolism during OA development.

## Conclusion

In conclusion, our study has clearly demonstrated that circPRKCH could act as a sponge of miR-145 and occupy an important position in promoting OA development by regulating chondrocyte apoptosis and ECM degradation via the HGF/c-MET signaling pathway. These findings demonstrated that circPRKCH might be utilized as a biomarker and target for OA therapy. Future in vivo and clinical research is required to identify the role of circPRKCH and miR-145 in OA progression.

## Supplementary Information

**Additional file 1: Supplementary Table 1.** Reagents and antibody information.

## Data Availability

The datasets used or analyzed during the current study are available from the corresponding author on reasonable request.

## Abbreviations

circRNAs: Circular RNAs
DMSO: Dimethyl sulfoxide
DMEM: Dulbecco’s modified Eagle medium
ECL: Enhanced chemiluminescence
ECM: Extracellular matrix
FBS: Fetal bovine serum
GAPDH: Glyceraldehyde-3-phosphate dehydrogenase
HE: Hematoxylin/eosin
HGF: Hepatocyte growth factor
HRP: Horseradish peroxidase
miRNAs: MicroRNAs
MMP-3: Matrix metalloproteinase 3
MMP-13: Matrix metalloproteinase 13
OA: Osteoarthritis
PI: Propidium iodide
U6: U6 small nuclear RNA
