# Supplementary material for: CircPRKCH modulates extracellular matrix formation and metabolism by regulating the miR-145/HGF axis in osteoarthritis
Source: Arthritis Res Ther. 2022 Sep 6;24:216. doi: 10.1186/s13075-022-02893-9 (PMC9447342; doi:10.1186/s13075-022-02893-9)
Supplement: Supplementary file 1 — Additional file 1: Supplementary Table 1. Reagents and antibody information. [file 13075_2022_2893_MOESM1_ESM.docx]

**Supplementary Table 1 Reagents and antibody information**

| **Reagents and antibodies** | **Catalog number** | **Company** | **Antibody clone number** |
| --- | --- | --- | --- |
| 0.25% trypsin | 25200056 | Invitrogen |  |
| 0.2% type II collagenase | 17101015 | Invitrogen |  |
| DMEM | 11966025 | Thermo Fisher Scientific |  |
| fetal bovine serum | 26140079 | Invitrogen |  |
| IL-1β | SRP6169 | Sigma |  |
| Lipofectamine 2000 reagent | [11668027](https://www.thermofisher.cn/order/catalog/product/cn/zh/11668027) | Invitrogen |  |
| puromycin | P8833 | Sigma |  |
| MTT | M5655 | Sigma |  |
| Annexin V-FITC/Propidium Iodide (PI) Apoptosis Kit | V13241 | Invitrogen |  |
| monosodium iodoacetate | 12512 | Sigma |  |
| Safranin O | HT90432 | Sigma |  |
| Fast Green | [F7252](https://www.sigmaaldrich.cn/CN/zh/product/sigma/f7252) | Sigma |  |
| Trizol | 15596018 | Invitrogen |  |
| PrimeScript™ RT Kit | RR037B | Takara |  |
| Mir-XTM miRNA First Strand Synthesis Kit | 638315 | Takara |  |
| SYBR Green PCR Kit | RR420L | Takara |  |
| RIPA | 89900 | Invitrogen |  |
| BCA protein assay kit | P0010 | Beyotime |  |
| anti-HGF | ab178395 | Abcam | EPR12230 |
| anti-MMP-3 | ab52915 | Abcam | EP1186Y |
| anti-MMP-13 | ab39012 | Abcam |  |
| anti-Aggrecan | ab3778 | Abcam | 6-B-4 |
| anti-Collagen Ⅱ | ab188570 | Abcam | EPR12268 |
| anti-c-MET | ab216574 | Abcam | EPR19067 |
| anti-c-MET(phospho Y1349) | ab68141 | Abcam | EP2367Y |
| GAPDH | ab8245 | Abcam | 6C5 |
| Rabbit Anti-Mouse IgG H&L (HRP) | ab6278 | Abcam |  |
| Goat Anti-Rabbit IgG H&L (HRP) | ab6721 | Abcam |  |
